# Awareness of Colorectal Cancer Risk Factors and Prevention-Related Beliefs Among Adults in Saudi Arabia: A Cross-Sectional Survey

**DOI:** 10.3390/healthcare14091244

**Published:** 2026-05-05

**Authors:** Arezki Azzi, Abdulrahman A. Aljaser, Ahmed H. Alassaf, Haifa H. Allahem, Naif A. Almansour, Sultan K. Alblaihi

**Affiliations:** 1 Department of Biochemistry, College of Medicine, Imam Mohammad Ibn Saud Islamic University (IMSIU), Riyadh 13317, Saudi Arabia; 2 Health Sciences Research Center (HSRC), Deanship of Scientific Research, Imam Mohammad Ibn Saud Islamic University (IMSIU), Riyadh 13317, Saudi Arabia; 3 College of Medicine, Imam Mohammad Ibn Saud Islamic University (IMSIU), Riyadh 13317, Saudi Arabia; 440012971@sm.imamu.edu.sa (A.A.A.); 440014208@sm.imamu.edu.sa (A.H.A.); 441019610@sm.imamu.edu.sa (H.H.A.); 440014901@sm.imamu.edu.sa (N.A.A.); 440017677@sm.imamu.edu.sa (S.K.A.)

**Keywords:** health beliefs, cancer prevention awareness, nutrition behaviors, exercise, body mass index, cross-sectional survey, Saudi Arabia

## Abstract

**Highlights:**

**What are the main findings?**

- Among Saudi adults surveyed, modifiable CRC risk factors and prevention were recognized in general, but important knowledge gaps persisted across specific behaviors.
- Overweight/obesity and suboptimal lifestyle patterns (diet and physical activity) were common, underscoring misalignment between awareness and practice.

**What are the implications of the main findings?**

- Culturally tailored education should focus on actionable prevention behaviors and improve understanding of key modifiable risks (e.g., weight control, physical activity, and diet quality).
- Public health strategies should target demographic groups with lower awareness or less favorable beliefs to support risk reduction and adoption of preventive behaviors.

**Abstract:**

**Background/Objectives**: Lifestyle factors (diet, physical activity, body weight) play a key role in colorectal cancer (CRC) prevention. This study assessed awareness, beliefs, and self-reported practices related to diet and physical activity for CRC prevention among adults living in Saudi Arabia, and examined demographic correlates. **Methods**: An analytic cross-sectional online survey was conducted among 544 adults (≥18 years). Measures included sociodemographics, self-reported height/weight (BMI), dietary and lifestyle practices, and beliefs about the role of a healthy diet and physical activity in cancer prevention. Associations were assessed using χ^2^ tests with effect size (Cramér’s V) and crude odds ratios (ORs) with 95% confidence intervals (CI); *p*-values < 0.001 are reported as <0.001. **Results**: Most participants reported consuming <3 daily servings of fruit (80.1%) and vegetables (69.9%). Overall, 41.9% of participants reported that their current fruit/vegetable intake did not meet minimum recommendations for cancer prevention, and 36.8% reported the same for physical activity. Overall, 80.1% believed that a healthy diet can help prevent cancer, and 79.4% believed that regular physical activity can help prevent cancer. Compared with females, males had higher odds of endorsing diet (OR 1.86, 95% CI 1.16–2.98) and physical activity (OR 1.98, 95% CI 1.24–3.17) as cancer-preventive. Participants with a monthly income of ≥5000 SAR had higher odds of endorsing diet (OR 2.71, 95% CI 1.75–4.19) and physical activity (OR 1.68, 95% CI 1.10–2.55) compared with those earning < 5000 SAR. **Conclusions**: Despite high belief in the preventive role of diet and physical activity, many participants reported suboptimal fruit/vegetable intake and insufficient activity. While these cross-sectional findings preclude causal inference, they suggest that health-promotion efforts may benefit from prioritizing lower-income groups and women, with an emphasis on practical approaches to meeting CRC-prevention lifestyle recommendations.

## 1. Introduction

Cancer is a broad term for many malignant types of neoplasms. One main concern with cancer is its ability to spread to distant sites from where it originated, making it harder to treat. One type of malignancy, and the focus of this study, is colorectal cancer (CRC), which is one of the most common malignancies worldwide and a leading cause of cancer mortality. Globally, CRC accounted for approximately 1.93 million new cases and 0.94 million deaths in 2020 [1]. In Saudi Arabia, CRC has shown a sustained increase in incidence over recent decades—with a 335.6% rise in absolute case numbers between 2001 and 2018, and CRC now constituting the most common cancer in Saudi males and the second or third most common in females [2,3,4,5].

The risk of CRC is strongly influenced by modifiable lifestyle factors, including diet quality, physical activity, adiposity, and smoking [6,7,8,9,10,11]. The World Cancer Research Fund/American Institute for Cancer Research (WCRF/AICR) and other expert bodies consistently recommend maintaining a healthy body weight, being physically active, and consuming a diet rich in fiber, whole grains, fruits, and vegetables while limiting red and processed meat, alcohol, and energy-dense foods [11,12].

National food-availability indicators suggest that common dietary staples in Saudi Arabia include meat and dairy products; for example, the General Authority for Statistics (GASTAT) reported a per capita total supply (available for consumption) of 10.80 kg/year of red meat and 70.19 L/year of milk in 2023 [13]. Understanding how these population patterns translate into individual practices and cancer-prevention awareness is important for targeting public health messaging.

Prevention of CRC is relevant to both clinical and public health settings: clinicians can use these findings to strengthen risk counseling during routine care, while public health agencies can use them to design population-level education and behavior-change strategies.

Although the role of lifestyle factors in CRC prevention is well established, awareness and implementation of recommendations may vary by gender, education, and socioeconomic status [14,15,16,17]. Evidence from Saudi Arabia indicates increasing prevalence of overweight/obesity and sedentary behaviors among adults [18,19,20], yet there is limited contemporary evidence describing how adults perceive diet and physical activity in relation to CRC prevention and whether these beliefs are associated with demographic characteristics. Across the broader Middle East and North Africa (MENA) region, CRC screening awareness remains low (6.5–38% in most countries surveyed), underscoring the importance of understanding public beliefs and behaviors that precede screening engagement [21].

This study aimed to describe self-reported dietary and physical activity practices relevant to CRC prevention among adults in Saudi Arabia and evaluate associations between demographic factors and beliefs about the role of a healthy diet and regular physical activity in cancer prevention. We hypothesized that higher education and higher income would be associated with stronger preventive health beliefs. While similar surveys have been conducted in other regions, population-specific data from Saudi Arabia documenting the gap between cancer-prevention beliefs and actual lifestyle behaviors, and their demographic correlates, remain limited. This study aims to address that gap by providing contemporary, region-specific evidence to inform targeted public health interventions in the Saudi context.

## 2. Materials and Methods

### 2.1. Study Design and Setting

Data collection was conducted from 21 May, 2024 to 2 August 2024 after approval from the institutional review board (IRB) of Imam Mohammad Ibn Saud Islamic University (IMSIU). Data were collected using an anonymous online questionnaire administered in Arabic.

### 2.2. Participants, Recruitment, and Sample Size

Inclusion criteria were: adults aged 18 years or older, currently residing in Saudi Arabia, able to read Arabic, and willing to provide electronic informed consent. Exclusion criteria were: age below 18 years, residence outside Saudi Arabia, and failure to provide informed consent. Survey responses with missing data on key variables (sex, age group, or primary belief outcomes) were additionally excluded from inferential analyses. The number of responses excluded due to missing key variables was small (*n* = 0 for the primary belief outcomes, as these were mandatory fields in the online form); therefore, complete-case analysis was applied, and no imputation was required. Participants were recruited through a non-probability convenience sampling approach via online dissemination of the survey link across public and institutional digital channels, including social media platforms (X [formerly Twitter]) and messaging applications (WhatsApp and Telegram). The survey link was shared by the research team and circulated through professional networks and university channels. The minimum required sample size (*n* = 385) was calculated using the Cochran formula for an infinite population (*p* = 0.5, q = 0.5, Z = 1.96, margin of error e = 5%), consistent with a 95% confidence level and a 5% margin of error applied to the adult Saudi population based on the latest national census. The final achieved sample was 544 participants.

### 2.3. Survey Instrument and Measures

The questionnaire collected: (a) sociodemographic characteristics (age group, sex, nationality, marital status, education, monthly income in Saudi Riyal [SAR]); (b) self-reported height and weight to compute body mass index (BMI, kg/m^2^); (c) lifestyle practices including dietary habits (frequency of red meat, dairy, eggs, fiber-rich foods, fruit and vegetable servings, and coffee intake), physical activity (frequency, duration, and intensity), sedentary time, smoking, alcohol intake, and use of selected medications/supplements (e.g., vitamin D). Belief outcomes included participants’ agreement that (a) eating a healthy diet can help prevent cancer and (b) regular physical activity can help prevent cancer, each with response options: Yes/No/Not sure. The questionnaire was developed by the research team based on established constructs from the literature on cancer prevention awareness and lifestyle behaviors. A pilot test was conducted with a small group of participants (*n* = 20) to evaluate comprehensibility and face validity; minor wording adjustments were made based on feedback before the final version was administered.

Personal or family history of colorectal cancer was collected as a potential correlate of prevention beliefs and was analyzed descriptively and in bivariate association analyses. The questionnaire also asked participants to judge whether their current diet and physical activity met minimum recommendations; however, it should be noted that the survey form did not provide detailed quantitative standards for cancer-prevention recommendations.

### 2.4. Statistical Analyses

Analyses were conducted using Statistical Packages for Social Sciences (SPSS) version 26 (IBM Corp., Armonk, NY, USA). Categorical variables are presented as counts and percentages. Associations between belief outcomes and demographic factors were examined using χ^2^ tests. Effect size was quantified using Cramér’s V. To improve interpretability, crude odds ratios (ORs) with 95% confidence intervals (CI) were calculated for selected comparisons by collapsing outcomes to Yes versus (No/Not sure). A two-sided *p*-value < 0.05 was considered statistically significant. *p*-values are presented to two decimal places, except when <0.001 or when close to 0.05 (0.045–0.054), in line with recommendations for reporting numerical data. Crude ORs use Yes vs. No/Not sure pairwise contrasts, while the *p*-values and Cramer’s V correspond to the overall three-category chi-square test. Because the available dataset consisted of self-reported cross-sectional survey responses and the present analysis was designed as exploratory and descriptive, we limited inference to bivariate analyses and explicitly acknowledge the absence of multivariable adjustment and sensitivity analyses as limitations.

### 2.5. Ethical Considerations

The IMSIU research ethics committee approved the study (project number 634-2024). The study was conducted according to the Declaration of Helsinki. Participants provided electronic informed consent prior to participation; no personally identifying information was collected.

## 3. Results

A total of 544 participants completed the survey (Table 1). Females represented 63.2% of the sample, and 40.4% were aged 18–30 years. Most participants were Saudi nationals (97.1%), and more than half were married (55.9%). Approximately two-thirds had a university degree (64.0%), and 43.4% reported a monthly income <5000 SAR. Based on self-reported height and weight, 64.5% were overweight or obese.

**Figure 1:**
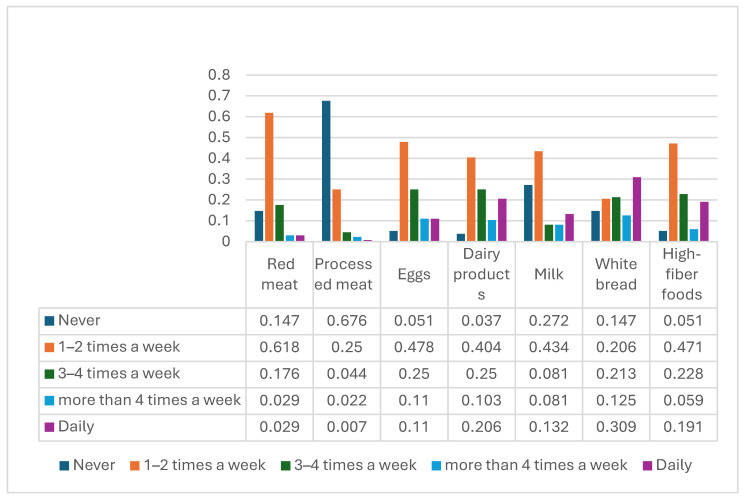
Frequency of consumption of selected food items relevant to CRC prevention. The figure summarizes reported intake frequency for red meat, processed meat, eggs, dairy products, milk, white bread, and high-fiber foods.

Dietary practices are summarized in Figure 1 and Table 2. Red meat was consumed once weekly by 61.8% of participants and 2–3 times weekly by 26.5%. Milk and dairy products were consumed daily by 31.4% of participants, and eggs were consumed 2–3 times weekly by 38.2%. Most participants reported consuming <3 daily servings of fruits (80.1%) and vegetables (69.9%), and 47.1% reported low intake of high-fiber foods.

Coffee consumption was common (Table 2): 43.4% reported drinking one cup of regular coffee per day, and 33.8% reported drinking 2–3 cups per day (Table 2). Most participants (75.7%) reported never consuming decaffeinated coffee. In terms of fruit intake, 80.1% of participants consumed less than three servings daily. Vegetable consumption followed a similar pattern, with 69.9% of participants eating less than three servings daily.

With respect to cancer-prevention recommendations, 41.9% reported that their current fruit and vegetable consumption did not meet minimum recommendations, and 36.8% reported that their physical activity did not meet minimum recommendations (Table 3). Many participants were unsure about both their diet and physical activity levels’ adequacy, with 39.0% and 41.9% expressing uncertainty, respectively. In terms of exercise frequency, 30.1% of participants reported no exercise days per week; 33.2% reported no exercise time on a given day. Among those who did exercise, 29.4% did so for less than 30 min per day. Regarding sedentary behavior, 39.7% of participants reported watching TV or playing games for fewer than 2 h per day, while 12.5% reported more than 6 h per day. The majority of participants described their physical activity as low intensity (71.3%). Regarding smoking habits, 77.9% of participants reported not smoking cigarettes. In terms of alcohol consumption, 96.3% of participants identified as either non-drinkers or past drinkers. Regarding medication usage, 64.8% did not take medications regularly, while 15.4% reported regular use of vitamin D supplements.

Table 4 summarizes associations between prevention-related beliefs and demographic factors. The *p*-values shown in Table 4 were obtained from Pearson chi-square tests comparing the full distribution of belief responses (Yes/No/Not sure) across categories of each demographic variable.

For diet-related beliefs, males were more likely than females to report that a healthy diet can help prevent cancer (86.0% vs. 76.7%; *p* = 0.03). Educational level, monthly income, and BMI category were also associated with this belief. Participants with a monthly income <5000 SAR were less likely than higher-income groups to endorse the cancer-preventive role of diet (71.2%; *p* < 0.001).

For physical-activity beliefs, males were also more likely than females to believe that regular physical activity can help prevent cancer (86.0% vs. 75.6%; *p* = 0.001). Marital status, educational level, monthly income, and BMI category showed significant associations, with the strongest gradients observed across income categories. These findings should be interpreted as associations rather than causal effects.

Personal or family history of CRC was reported by 12.5% of participants and, in bivariate analyses, was not significantly associated with belief that diet or physical activity can help prevent cancer (Table 4).

Table 5 presents crude (unadjusted) odds ratios (OR) for the belief that diet and physical activity can prevent cancer, across several demographic predictors. The table compares the likelihood of answering “Yes” versus “No/Not sure” across different groups. In χ^2^ analyses, belief in the cancer-preventive role of a healthy diet differed by sex (*p* = 0.03; Cramér’s V = 0.11), marital status (*p* = 0.03; V = 0.11), educational level (*p* = 0.001; V = 0.15), monthly income (*p* < 0.001; V = 0.33), and BMI category (*p* = 0.001; V = 0.15), but not by age group or nationality. Belief in the cancer-preventive role of physical activity differed by sex (*p* = 0.001; V = 0.16), marital status (*p* < 0.001; V = 0.26), educational level (*p* < 0.001; V = 0.37), monthly income (*p* < 0.001; V = 0.51), and BMI category (*p* = 0.02; V = 0.12). In crude OR analyses (Yes vs. No/Not sure), males had higher odds than females of endorsing diet (OR 1.86, 95% CI 1.16–2.98) and physical activity (OR 1.98, 95% CI 1.24–3.17) as cancer-preventive. Monthly income ≥5000 SAR was associated with higher odds of endorsing diet (OR 2.71, 95% CI 1.75–4.19) and physical activity (OR 1.68, 95% CI 1.10–2.55) compared with income <5000 SAR. Personal or family history of colorectal cancer was not included in the selected crude OR comparisons because no statistically significant association was observed in the overall chi-square tests.

## 4. Discussion

We examined lifestyle practices related to CRC prevention and the demographic correlates of beliefs about the preventive roles of diet and physical activity among adults in Saudi Arabia. A clear belief–behavior gap was evident in this sample. Although a large majority endorsed healthy diet and regular physical activity as cancer-preventive, substantial proportions reported not meeting minimum lifestyle recommendations (particularly fruit/vegetable intake and physical activity). Socioeconomic variables (especially income) showed the strongest associations with preventive beliefs.

### 4.1. Demographic Overview

The sample was predominantly female (63.2%) and relatively young, with 40.4% aged 18–30 years, with high educational attainment, as 64.0% had attained a university degree. The prevalence of obesity (32.0%) and overweight (32.5%) among participants is similar to what has been reported in several previous studies [18,19,20].

### 4.2. Dietary Habits, Physical Activity, and Lifestyle Behaviors

Participants commonly reported dietary patterns that do not align well with CRC-prevention guidance, particularly low fruit, vegetable, and high-fiber intake. The dietary habits of participants revealed a high consumption of red meat, dairy products, and white bread, with 61.8% consuming red meat 1–2 times per week and 30.9% consuming white bread daily. These consumption patterns are concerning, as many previous studies show that a high intake of red meat and low fiber intake are associated with an increased cancer risk [22,23,24]. Population-level food-availability indicators in Saudi Arabia also show notable per capita supply of animal products (e.g., red meat 10.80 kg/year; milk 70.19 L/year in 2023) [13]. The frequency of fruit and vegetable consumption was below recommended levels, with 80.1% of participants eating less than three servings of fruit daily and 69.9% consuming fewer than three servings of vegetables. These findings are concerning because international evidence consistently links lower diet quality (low fiber, whole grains, and fruit/vegetable intake; high red/processed meat and energy-dense food consumption) with higher CRC risk and other chronic diseases [25,26,27]. A 2025 Global Cancer Update Programme (CUP Global) systematic review found strong-probable evidence that greater alignment with WCRF/AICR dietary-lifestyle recommendations is associated with lower CRC risk [28], reinforcing the clinical significance of the suboptimal dietary patterns documented in our sample. Coffee consumption was common in this sample. Coffee consumption patterns showed a preference for regular coffee over decaffeinated coffee, with 43.4% drinking one cup of regular coffee daily. This preference for regular coffee is consistent with trends observed in other populations [14,15]. Systematic reviews reported that coffee drinking was associated with a lower risk of CRC in several analyses, although the certainty of evidence varies across reviews [29]. Public health messaging should present coffee as a potential contributor within an overall healthy dietary pattern.

However, the high proportion of participants consuming less than the recommended amount of fruits and vegetables and the low consumption of high-fiber foods indicate potential areas for dietary improvement.

Only 15.4% of participants reported vitamin D supplementation. Given observational associations between vitamin D status and lower CRC risk [30,31], future work should assess vitamin D levels and supplementation practices in this population.

Low rates of smoking (77.9% non-smokers) and alcohol use (96.3% non- or past drinkers) represent favorable behavioral profiles in this sample. Only 15.4% reported vitamin D supplementation, which is noteworthy given the established observational association between vitamin D status and CRC risk [30,31,32].

### 4.3. Beliefs and Attitudes Toward Cancer Prevention

Preventive beliefs differed by sex, education, income, and BMI. Males were more likely to believe in the cancer-preventive benefits of both diet and physical activity compared to females, a finding that echoes previous research showing gender differences in health beliefs [16,17]. Educational level had a strong influence on health beliefs, with participants holding university degrees demonstrating greater beliefs in the benefits of a healthy diet and physical activity. This finding supports existing literature that associates higher education with increased health literacy and adherence to preventive health behaviors [33,34]. Income showed the largest effect sizes (Cramér’s V up to 0.51 for physical activity belief), suggesting a strong socioeconomic gradient in preventive health beliefs. This may reflect differences in access to health information, health literacy, and the perceived affordability of healthy foods and structured physical activity. These findings support targeted interventions that prioritize lower-income groups and women, using practical strategies to translate awareness into daily behaviors. The strong income gradient observed in our data aligns with broader evidence that socioeconomic status is a robust determinant of cancer-preventive behaviors and cancer outcomes across populations [35], reinforcing the need for equity-focused public health approaches in the Saudi context.

### 4.4. BMI and Health Beliefs

Normal-weight participants were more likely to endorse the cancer-preventive benefits of diet and physical activity than those who were overweight or obese, suggesting a potential positive feedback between healthy weight status and health-promoting beliefs. Weight management programs may therefore benefit from integrating cancer-prevention education [36].

### 4.5. Physical Activity and Sedentary Behavior

A substantial proportion of participants did not meet the recommended levels of physical activity: 30.1% reported no exercise days per week, and 29.4% exercised for less than 30 min per day. These patterns of low physical activity and high sedentary behavior are concerning, as they are associated with an increased cancer risk [12,37,38,39]. Most participants described their physical activity as low-intensity, which may not confer the same health benefits as moderate- or high-intensity activity. Interpretation of sex differences in physical-activity beliefs and practices should consider contextual barriers and opportunities for women in Saudi Arabia, including variability in access to safe walkable environments, women-only facilities, transportation, cost, and time constraints related to work and caregiving responsibilities. These factors may influence both perceived feasibility of exercise and engagement in regular activity, and should be considered when designing culturally appropriate interventions.

### 4.6. Implications for Public Health

These findings underscore the need for demographic-tailored public health strategies. Interventions targeting lower-income groups and women, who showed weaker preventive beliefs and less favorable lifestyle profiles, should emphasize practical, accessible ways to adopt cancer-prevention behaviors in culturally appropriate contexts. Integration of CRC-prevention messaging into primary care and community health settings may further help translate awareness into action. Given that CRC screening uptake in Saudi Arabia remains very low (estimated at 4.2% in primary care settings) [21], lifestyle-focused education must be coupled with expanded and accessible screening programs to achieve meaningful reductions in CRC incidence and mortality.

### 4.7. Strengths and Limitations

Strengths of this study include the assessment of multiple lifestyle dimensions relevant to CRC prevention and the addition of effect sizes and ORs to support interpretation. However, several limitations should be considered: the cross-sectional design precludes causal inference; all measures were self-reported (including BMI); the online recruitment approach may introduce selection bias toward younger and more educated participants. Additional limitations are that key comorbidities associated with CRC risk (e.g., inflammatory bowel disease, type 2 diabetes/metabolic disorders, and prior colorectal polyps) were not measured, and some uncertainty in participants’ self-assessment of whether they met minimum diet or physical-activity recommendations may reflect the absence of detailed quantitative standards in the survey form. Finally, the study evaluates beliefs and behaviors related to CRC prevention rather than CRC incidence; therefore, results should be interpreted as describing awareness and practices, not disease risk. Regarding missing data, all primary belief outcome items were mandatory fields in the online survey form; thus, complete responses were obtained for these variables, and complete-case analysis was appropriate. A small number of responses with missing values for key demographic variables (sex or age group) were excluded from inferential analyses. Because these exclusions were rare, they are unlikely to have meaningfully affected the results.

## 5. Conclusions

In this cross-sectional survey of adults in Saudi Arabia, most participants endorsed diet and physical activity as cancer-preventive; however, many reported suboptimal fruit/vegetable intake and insufficient physical activity, reflecting a gap between belief and practice. Preventive health beliefs were strongly patterned by income and, to a lesser extent, by sex and education. These findings should be interpreted cautiously, given the cross-sectional design and reliance on self-reported measures, which preclude causal inference. Future research incorporating objective measures and longitudinal or interventional designs is needed to confirm these associations. Nonetheless, the results support targeted public health programs that prioritize practical, accessible behavior-changing strategies for lower-income groups and women, and that integrate locally relevant dietary guidance and culturally appropriate opportunities for physical activity.

## Figures and Tables

**Table 1:** Demographic factors of the participants.

		Count	Column *N*%
Gender	Male	200	36.8
	Female	344	63.2
Age [years]	18–30	220	40.4
	31–45	100	18.4
	46–60	216	39.7
	61–80	8	1.5
Nationality	Saudi	528	97.1
	Non-Saudi	16	2.9
Marital status	Single	212	39.0
	Married	304	55.8
	Divorced	20	3.7
	Widow	8	1.5
Educational level	Primary school	4	0.7
	High school	104	19.1
	Diploma	48	8.8
	University	348	64.0
	Post-graduated	40	7.4
Monthly income	<5000 SAR	236	43.3
	5001–10,000 SAR	132	24.3
	10,001–20,000 SAR	140	25.7
	20,001–30,000 SAR	20	3.7
	30,001–50,000 SAR	8	1.5
	>50,000 SAR	8	1.5
Do you have a Personal history or a family history of Colorectal Cancer	No	476	87.5
	Yes	68	12.5
BMI	Underweight	24	4.4
	Normal weight	169	31.1
	Overweight	177	32.5
	Obese	174	32.0

Note: Monthly income is reported in Saudi Riyal (SAR). For international readers, the currency is pegged at 1 USD = 3.75 SAR.

**Table 2:** Dietary intake frequency: selected food and beverage items.

		Count	Column *N*%
How many cups of regular coffee (not decaf) do you usually drink each day?	Never	52	9.6
	1 cup	236	43.4
	2–3 cups	184	33.8
	4–5 cups	44	8.1
	6 or more cups	28	5.1
How many cups of Decaffeinated coffee do you usually drink each day?	Never	412	75.7
	1 cup	80	14.7
	2–3 cups	48	8.8
	4–5 cups	4	0.7
	6 or more cups	0	0.0
How often do you consume fruits in your diet?	Less than three servings daily	436	80.1
	From 2 to 4 servings daily	92	16.9
	More than 6 servings daily	16	2.9
How often do you consume vegetables in your diet?	Less than three servings daily	380	69.8
	From 2 to 4 servings daily	144	26.5
	More than 6 servings daily	20	3.7

**Table 3:** Self-reported cancer-prevention beliefs and physical activity characteristics.

		Count	Column *N*%
Do you believe that your Current fruit/vegetable consumption meets minimum recommendations for cancer prevention?	No	228	41.9
	Yes	104	19.1
	Not sure	212	39.0
Do you believe that your current level of physical activity meets the minimum recommendation for cancer prevention?	No	200	36.8
	Yes	116	21.3
	Not sure	228	41.9
How many days per week do you practice exercise?	None	164	30.1
	1	108	19.9
	2	72	13.2
	3	76	14.0
	4	40	7.4
	5	60	11.0
	6	20	3.7
	7	4	0.7
How much time per day do you exercise?	Never	180	33.2
	Less than 30 min	160	29.4
	30 min–1 h	116	21.3
	1–2 h	84	15.4
	More than 2 h	4	0.7
How many hours do you sit down watching TV or playing games per day?	Less than 2 h	216	39.7
	between 2 and 4 h	184	33.8
	4 to 6 h	76	14.0
	More than 6 h	68	12.5
How would you describe the intensity of your physical activity?	Low intensity (leisurely walking, stretching exercises, household chores)	388	71.3
	Moderate intensity (cycling, swimming, brisk walking)	116	21.3
	High intensity (aerobic exercise, intense sports, running)	40	7.4
Do you smoke cigarettes?	No	424	77.9
	Yes	68	12.5
	Other forms of smoking (vaping, e-cigarettes, shisha)	52	9.6
Do you consume alcohol?	NO/PAST drinker.	524	96.3
	<1 drink per day	20	3.7
	>1 drink per day	0	0.0
Do you take these medications regularly?	No	352	64.8
	Yes, aspirin.	20	3.7
	Yes, folic acid	4	0.7
	Yes, vitamin D.	84	15.4
	Yes, non-steroidal anti-inflammatory drugs (ex. Ibuprofen).	12	2.2
	Yes, a cholesterol-reducing drug	72	13.2

**Table 4:** Associations between prevention-related beliefs and demographic factors.

		Do You Believe Eating a Healthy Diet Can Help Prevent Cancer?						*p*-Value	Do You Believe Regular Physical Activity Can Help Prevent Cancer?						*p*-Value
		No		Yes		Not Sure			No		Yes		Not Sure		
		Count	Row *N*%	Count	Row *N*%	Count	Row *N*%		Count	Row *N*%	Count	Row *N*%	Count	Row *N*%	
Gender	Male	4	2.0	172	86.0	24	12.0	0.03 *	8	4.0	172	86.0	20	10.0	0.001 *
	Female	12	3.5	264	76.7	68	19.8		8	2.3	260	75.6	76	22.1	
Age	18–30	4	1.8	176	80.0	40	18.2	0.50	4	1.8	184	83.6	32	14.5	0.30
	31–45	4	4.0	76	76.0	20	20.0		4	4.0	76	76.0	20	20.0	
	46–60	8	3.7	176	81.5	32	14.8		8	3.7	164	75.9	44	20.4	
	61–80	0	0.0	8	100.0	0	0.0		0	0.0	8	100.0	0	0.0	
Nationality	Saudi	16	3.0	424	80.3	88	16.7	0.56	16	3.0	420	79.5	92	17.4	0.60
	Non-Saudi	0	0.0	12	75.0	4	25.0		0	0.0	12	75.0	4	25.0	
Marital status	Single	4	1.9	168	79.2	40	18.9	0.03 *	4	1.9	176	83.0	32	15.1	<0.001 *
	Married	12	3.9	244	80.3	48	15.8		8	2.6	232	76.3	64	21.1	
	Divorced	0	0.0	20	100.0	0	0.0		0	0.0	20	100.0	0	0.0	
	Widow	0	0.0	4	50.0	4	50.0		4	50.0	4	50.0	0	0.0	
Educational level	Primary school	0	0.0	4	100.0	0	0.0	0.001 *	4	100.0	0	0.0	0	0.0	<0.001 *
	High school	0	0.0	76	73.1	28	26.9		0	0.0	80	76.9	24	23.1	
	Diploma	4	8.3	36	75.0	8	16.7		0	0.0	40	83.3	8	16.7	
	University	8	2.3	288	82.8	52	14.9		8	2.3	280	80.5	60	17.2	
	Post-graduated	4	10.0	32	80.0	4	10.0		4	10.0	32	80.0	4	10.0	
Monthly income	<5000 SR	4	1.7	168	71.2	64	27.1	<0.001 *	4	1.7	176	74.6	56	23.7	<0.001 *
	5001–10,000	8	6.1	100	75.8	24	18.2		4	3.0	104	78.8	24	18.2	
	10,001–20,000	0	0.0	136	97.1	4	2.9		0	0.0	124	88.6	16	11.4	
	20,001–30,000	0	0.0	20	100.0	0	0.0		0	0.0	20	100.0	0	0.0	
	30,001–50,000	0	0.0	8	100.0	0	0.0		0	0.0	8	100.0	0	0.0	
	>50,000 SR	4	50.0	4	50.0	0	0.0		8	100.0	0	0.0	0	0.0	
Do you have a Personal history or a family history of Colorectal Cancer	No	12	2.5	380	79.8	84	17.6	0.17	12	2.5	376	79.0	88	18.5	0.14
	Yes	4	5.9	56	82.4	8	11.8		4	5.9	56	82.4	8	11.8	
BMI	Underweight	0	0.0	24	100.0	0	0.0	0.001 *	0	0.0	20	83.3	4	16.7	0.02 *
	Normal weight	4	2.4	141	83.4	24	14.2		0	0.0	145	85.8	24	14.2	
	Overweight	12	6.8	133	75.1	32	18.1		8	4.5	141	79.7	28	15.8	
	Obese	0	0.0	138	79.3	36	20.7		8	4.6	126	72.4	40	23.0	

* Significant at *p* < 0.05 level.

**Table 5:** Crude odds ratios (OR) for endorsing diet and physical activity as cancer-preventive (Yes vs. No/Not sure).

Predictor	Comparison	Outcome	OR (95% CI)	χ^2^ *p*-Value	Cramér’s V
Gender	Male vs. Female	Belief: healthy diet prevents cancer	1.86 (1.16–2.98)	0.03	0.11
Gender	Male vs. Female	Belief: physical activity prevents cancer	1.98 (1.24–3.17)	0.001	0.16
Education	University/Postgrad vs. ≤Diploma	Belief: healthy diet prevents cancer	1.62 (1.04–2.53)	0.001	0.15
Education	University/Postgrad vs. ≤Diploma	Belief: physical activity prevents cancer	1.23 (0.79–1.93)	<0.001	0.37
Monthly income	≥5000 SAR vs. <5000 SAR	Belief: healthy diet prevents cancer	2.71 (1.75–4.19)	<0.001	0.33
Monthly income	≥5000 SAR vs. <5000 SAR	Belief: physical activity prevents cancer	1.68 (1.10–2.55)	<0.001	0.51
BMI	Normal vs. Overweight/Obese	Belief: healthy diet prevents cancer	1.49 (0.92–2.39)	<0.001	0.15
BMI	Normal vs. Overweight/Obese	Belief: physical activity prevents cancer	1.90 (1.16–3.12)	0.02	0.12
Age	18–30 vs. ≥46	Belief: healthy diet prevents cancer	0.87 (0.54–1.40)	0.50	0.07
Age	18–30 vs. ≥46	Belief: physical activity prevents cancer	1.55 (0.96–2.48)	0.30	0.08
Marital status	Married vs. Not married	Belief: healthy diet prevents cancer	1.02 (0.67–1.55)	0.03	0.11
Marital status	Married vs. Not married	Belief: physical activity prevents cancer	0.64 (0.42–0.99)	<0.001	0.26

Note: ORs are crude (unadjusted). χ^2^ *p*-values and Cramér’s V correspond to the overall association between each predictor and the three-level belief response (Yes/No/Not sure).

## Data Availability

The original contributions presented in this study are included in the article. Further inquiries can be directed to the corresponding authors. The dataset cannot be made publicly available because it contains sensitive human-subject information and is restricted under ethical requirements and regulations of the IMSIU Institutional Review Board (IRB) that approved the study.
